# Preventive Effect of Phytic Acid on Isoproterenol-Induced Cardiotoxicity in Wistar Rats

**Published:** 2015-03

**Authors:** E. Brindha, M. Rajasekapandiyan

**Affiliations:** 1 Department of Biotechnology, Muthaymmal college of Arts and Science, Rasipuram, Namakkal DT, Tamilnadu, India;; 2 Department of Zoology, Arignar Anna Government Arts and Science college, Namakkal DT, Tamilnadu, India

**Keywords:** Phytic acid, Isoproterenol, Myocardial infarction, Glycoproteins, Membrane bound enzymes

## Abstract

This study was aimed to evaluate the preventive role of phytic acid on membrane bound enzymes such as sodium potassium- dependent adenosine triphosphatase (Na^+^ /K^+^ ATPase), calcium-dependent adenosine triphosphatase (Ca^2+^ ATPase) and magnesium- dependent adenosine triphosphatase (Mg^2+^ ATPase) and glycoproteins such as hexose, hexosamine, fucose and sialic acid in isoproterenol (ISO)-induced myocardial infarction (MI) in rats. Male albino Wistar rats were pretreated with phytic acid (25 and 50 mg/kg, respectively) for a period of 56 days. After the treatment period, ISO (85 mg/kg) was subcutaneously injected to rats at an interval of 24 h for 2 days. ISO-induced rats showed a significant decrease in the activity of Na^+^ /K^+^ ATPase and increase in the activities of Ca^2+^ and Mg^2+^ ATPase in the heart and a significant (*P*<0.05) increase in the levels of glycoproteins in serum and the heart were also observed in ISO-induced rats. Pretreatment with phytic acid for a period of 56 days exhibited a significant (*P*<0.05) effect and altered these biochemical parameters positively in ISO-induced rats. Thus, our study shows that phytic acid has cardioprotective role in ISO-induced MI in rats.

## INTRODUCTION

Cardiovascular diseases are the leading cause of death in the world. The cardiovascular system is susceptible to many chronic diseases such as hypertension and myocardial infarction (MI). Damage to the myocardial cells arises due to the generation of toxic reactive oxygen species (ROS) such as superoxide radicals, H_2_O_2_ and hydroxyl radical (1). It is well known that cardiovascular disease (CVD) is directly or indirectly related to oxidative damage that shares a common mechanism of molecular and cellular damage. Auto-oxidation of catecholamines results in the generation of highly cytotoxic free radicals (2). Free radicals could initiate the peroxidation of membrane bound polyunsaturated fatty acids (PUFA), leading to both functional and structural myocardial injury (3). The effects of isoproterenol (ISO) on heart are mediated through β_1_ and β_2_ adrenoceptors. Both β_1_ and β_2_ adrenoceptors mediate the positive inotropic and chronotropic effects to β adrenoceptor agonists (4). Thus, ISO produces relative ischemia or hypoxia due to myocardial hyperactivity and coronary hypotension (5) and induce myocardial ischemia due to cytosolic Ca^2+^ overload (6). Grimm *et al*. (7) have reported that a toxic dosage of ISO caused characteristic myocardial damage that subsequently resulted in heart failure. ISO-administration causes ischemic necrosis in rats, which closely resembles histological damage seen in human MI. The functions of glycoproteins in stabilizing the tissue may be involved in maintaining the structural stability of collagen fibrils. Glycoproteins are important components of intracellular matrix, cell membrane and membranes of the subcellular organelles (8). ATPases are intimately associated with the plasma membrane and participates in the energy requiring translocation of sodium, potassium, calcium and magnesium (9). The inhibition of sodium potassium dependent adenosine triphosphatase (Na^+^/K^+^-ATPase) can activate the Na^+^-Ca^2+^ exchange mechanism in the myocardium. This Na^+^-Ca^2+^ exchange mechanism may play a vital role in regulating the cellular calcium levels (10). Calcium dependent adenosine triphosphatase (Ca^2+^-ATPase) is the major active calcium transport protein responsible for the maintenance of normal intracellular calcium levels in a variety of cell types.

Phenolic compounds form a substantial part of plant foods and a vital role in the development of various human diseases including CVDs. Most of these phenolic compounds are antioxidants *in vitro* and antioxidants may protect against CVDs (11). Phytic acid (myo-inositol hexaphosphate, IP6) is widely found in cereals, nuts, leguumes, oil seeds, pollen, and spores. Structural studies established that it contains phosphorous, which binds minerals such as calcium, iron, and zinc causing a decrease of their bioavailability in human and animal model (12). However, recently, phytic acid has been reported to be an antioxidant (Graf and Eaton, 1990), anticarcinogenic (13) and hypoglycemic or hypolipidemic (14). Phenolic acids have received much attention because of their role in the prevention of many human diseases, particularly atherosclerosis and cancer due to their antioxidant properties (15). Very few reports are available on the effect of nonflavonoids such as phenolic acids and their mechanism in MI. A previous scientific report has shown that phytic acid inhibits oxidation of low-density lipoprotein *in vitro* and might therefore protect against CVDs (16). The mechanism of action of ISO-induced MI is not clearly understood. Biological compounds with antioxidant properties contribute o the protection of cells and tissues against deleterious effects of reactive oxygen species and other free radicals (17). There are no *in vivo* studies available on the effect of phytic acid in MI. Therapeutic intervention that could improve impaired antioxidant defense mechanism or diminish free radical production in the ischemic myocardium has been of great interest. By decreasing free radical production, one can prevent cardiac tissue damage and protect the heart from free radical-mediated cardiac tissue damage and protect the heart from free radical production in the ischemic myocardium has been of great interest. By decreasing free radical production, one can prevent cardiac tissue damage and protect the heart from free radical – mediated cardiac damage in ISO- induced rats.

## MATERIALS AND METHODS

### Experimental Animals

The experiment was carried out according to the guidelines of the Committee for the Purpose of Control and Supervision of Experiments on Animals (CPCSEA), New Delhi, India and approved by the Animal Ethical Committee of Bharathidasan University (Approval no.BDU/IAEC/2011/31/29.03.2011). All the experiments were carried out with male albino Wistar rats weighing 140-160 g, obtained from the Central Animal House, Rajah Muthiah Institute of Health Sciences, Annamalai University, Annamalai Nagar, Tamil Nadu, India. They were housed in polypropylene cages (47 × 34 × 20 cm) lined with husk, renewed every 24 h under 12:12 h light dark cycle at around 22°C and had free access to tap water and food. The rats were fed on a standard pellet diet. (Pranav Agro Industries Ltd., Maharashtra, Pune, India). The pellet diet consists of 21% protein, 5% lipids, 4% crude fiber, 8% ash, 1% calcium, 0.6% phosphorus, 3.4% glucose, 2% vitamins and 55% nitrogen free extract. The diet provides metabolizable energy of 3,600 kcal.

### Drugs and chemicals

Isoproterenol hydrochloride, phytic acid, trichloroacetic acid (TCA), galactose, thiobarbituric acid (TBA), *p*-dimethyl amino benzaldehyde, and acetyl acetone reagent were purchased from Sigma Chemical Company, St. Louis, MO, USA. Glucose, uric acid, total protein and A/G ratio kits were purchased from Qualigens Diagnostics, Mumbai, India. All other chemicals used in this study were of analytical grade.

### Induction of experimental myocardial infarction

Isoproterenol (85 mg/kg) was dissolved in normal saline and injected subcutaneously to rats at an interval of 24 h for 2 days (19).

### Experimental design

The rats were grouped as 10 rats in each group. Group 1: Normal control rats; Group 2: normal rats treated with phytic acid (25 mg/kg); Group 3; normal rats treated with phytic acid (50 mg/kg); group4; ISO (85 mg/kg) control rats; groups 5 rats pretreated with phytic acid (25 mg/kg) and groups 6 rats pretreated with phytic acid (50 mg/kg) then subcutaneously injected with ISO. Phytic acid was dissolved in distilled water and administered to rats orally using an intragastric tube daily for a period of 56 days.

At the end of the experimental period, after 12 h of the second ISO-injection, all the rats were anesthetized with sodium pentobarbital and killed by cervical decapitation. The heart tissue was excised immediately from the animals, and blood washed off with ice-chilled physiological saline.

### Biochemical assays

The tissue samples were defatted prior to estimation. The tissue homogenate were defatted by the method of Folch *et al* (20), using chloroform–methanol mixture (CHCl3:MeOH) (2:1 v/v). A known weight of heart tissue was homogenized in 7 ml of methanol using Potter-Elvehjam homogenizer. The contents were filtered into a previously weighed side arm flask and the residue on the filter paper was scrapped off and homogenized with 14 ml of CHCl3:MeOH mixture. This was again filtered into the side-arm flask and the residue was successively homogenized in CHCl3:MeOH (2:1 v/v) and each time this extract was filtered. The pooled filtrates in the flask were adjusted to a final volume ratio using CHCl3:MeOH (2:1 v/v) and evaporated to dryness to a constant weight. A weighed amount of defatted tissue was suspended in 3.0 ml of 2 N HCl and heated at 90 C for 4 hr. The sample was cooled and neutralized with 3.0 ml of 2 N NaOH. Aliquots from this were used for estimation of hexose, hexosamine, sialic acid and fucose.

Protein-bound hexose was estimated by the method of Dubois and Gilles (18). To 0.1 ml of sample, 5.0 ml of 95% ethanol was added, mixed and then centrifuged. The precipi- tate was dissolved in 1.0 ml of 0.1 N NaOH. To all the tubes, 8.5 ml of orcinol–sulfuric acid reagent was added and kept in a water bath at 90°C for 15 min. The tubes were cooled in tap water and the colour developed was read at 540 nm.

Protein bound hexosamine was estimated according to the method of Wagner (21). To 1.0 ml of sample, added 2.5 ml of 3 N HCl and kept for 6 h in a boiling water bath and then neutralized with 6 N NaOH. To 0.8 ml of the neutralized sample, added 0.6 ml of acetyl acetone reagent. Then, the tubes were heated in a boiling water bath for 30 min. After cooling, 2.0 ml of Ehrlich’s reagent was added and mixed well. The intensity of the colour developed was read at 540 nm.

Fucose was estimated by the method of Dische and Shettles (22). To 0.5 ml of sample, 4.5 ml of H_2_SO_4_ was added and heated in a boiling water bath for 3 min, cooled and 0.1 ml of cysteine reagent was added. After 75 min in dark, the absorbance was read at 393 and 430 nm. The fucose content was calculated from the difference in the readings obtained at 393 and 430 nm and then the values were substracted to obtain values excluding cysteine.

Sialic acid was estimated by the method of Warren (23). To 0.5 ml of sample, 0.2 ml of distilled water and 0.25 ml of periodic acid were added and incubated at 37°C for 30 min. To this, 0.2 ml of sodium meta arsenate and 2.0 ml of TBA were added and heated in a boiling water bath for 6 min, cooled and 5.0 ml of acidified butanol was added. The absorbance was read at 540 nm. The activity of Na^+^ /K^+^ ATPase was assayed according to the procedure of Bonting (24). The incubation mixture con- tained 1.0 ml of buffer, 0.2 ml of magnesium sulphate, 0.2 ml of potassium chloride, 0.2 ml of sodium chloride, 0.2 ml of EDTA, 0.2 ml of ATP and 0.2 ml of tissue homogenate. The contents were incubated at 37°C for 15 min. A 1.0 ml of ice-cold 10% TCA was added at the end of 15 min to arrest the reaction. The amount of phosphorus liberated was estimated as described by Fiske and Subbarow (25). A 1.0 ml of the supernatant was made up to 4.0 ml with distilled water and 1.0 ml of 2.5% ammonium molybdate was added. This was incubated at room temperature for 10 min and 0.4 ml of aminonapthol sulfonic acid was added. The colour developed was read spectrophoto- metrically at 640 nm after 20 min.

The activity of Ca^2+^ ATPase was assayed according to the method of Hjerten and Pan (26). The incubation mixture contained 0.1 ml of buffer, 0.1 ml of calcium chloride, 0.1 ml of ATP, 0.1 ml of distilled water and 0.1 ml of tissue homogenate.

The contents were incubated at 37°C for 15 min. The reaction was then arrested by the addition of 0.5 ml of ice-cold 10% TCA. The amount of phosphorus liberated was estimated according to the method of Fiske and Subbarow (25).

The activity of Mg^2+^ ATPase was assayed according to the method of Ohnishi *et al*. (27). The incubation mixture con- tained 0.1 ml of buffer, 0.1 ml of magnesium chloride, 0.1 ml of ATP, 0.1 ml of distilled water and 0.1 ml of tissue homogenate. The reaction mixture was incubated at 37°C for 15 min. The reaction was arrested by the addition of 0.5 ml of ice cold 10% TCA. The amount of phosphorous liberated was estimated by the method of Fiske and Subbarow (25).

### Statistical analysis

Statistical analysis was performed using one-way analysis of variance (ANOVA) followed by Duncan’s Multiple Range Test (DMRT) using Statistical Package form the Social Sciences (SPSS) software package Version 9.05. Results were expressed as mean ± S.D. for six rats in each group. *P*-value<0.05 were considered as significant.

## RESULTS

**Figure 1:**
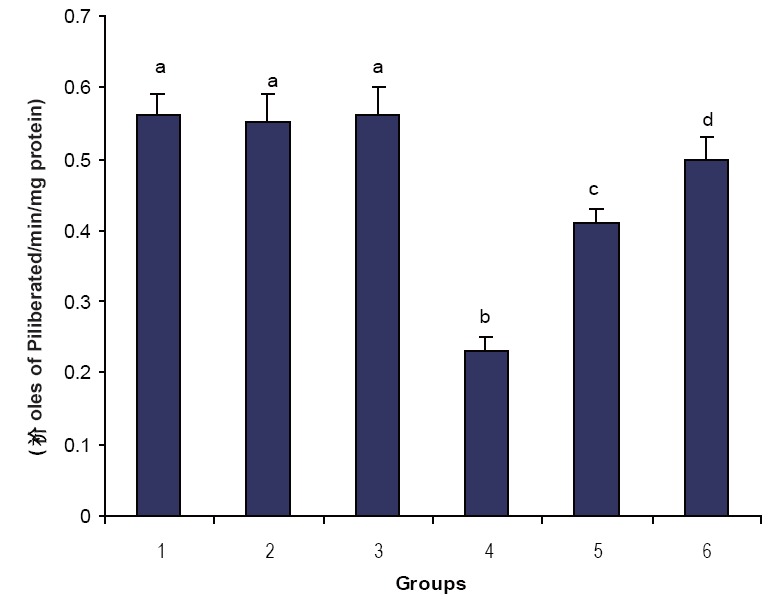
Effect of Phytic acid on the activity sodium potassium dependent adenosine triphosphatase (Na^+^/K^+^ ATPase) in the heart of normal and isoproterenol (ISO)- induced myocardial infracted rats. Group 1: normal control; Groups 2-3: Normal + phytic acid (25 and 50 mg/kg); Group 4: ISO control (85 mg/kg); Group 5-6: phytic acid (25 and 50 mg/kg) + ISO. Each value is mean ± S.D. for 6 rats in each group. Columns not sharing a common letter (a, b, c and d) differ significantly with each other (*p*<0.05, DMRT).

**Figure 2:**
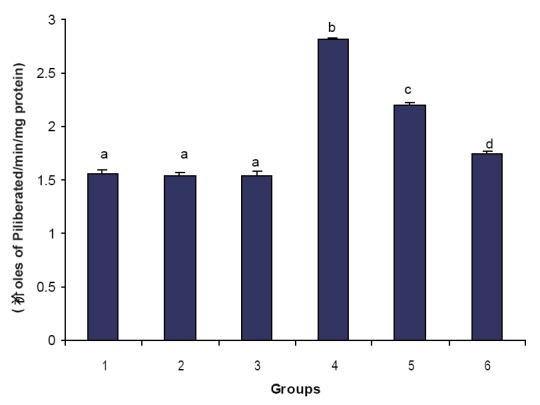
Effect of phytic acid on the activity of calcium dependent adenosine triphosphatase (Ca^2+^ -ATPase) in the heart of normal and isoproterenol (ISO)-induced myocardial infracted rats. Group 1: normal control; Groups 2-3: Normal + phytic acid (25 and 50 mg/kg); Group 4: ISO control (85 mg/kg); Group 5-6: phytic acid (25 and 50 mg/kg) + ISO. Each value is mean ± S.D. for 6 rats in each group. Columns not sharing a common letter (a, b, c and d) differ significantly with each other (*p*<0.05, DMRT).

**Figure 3:**
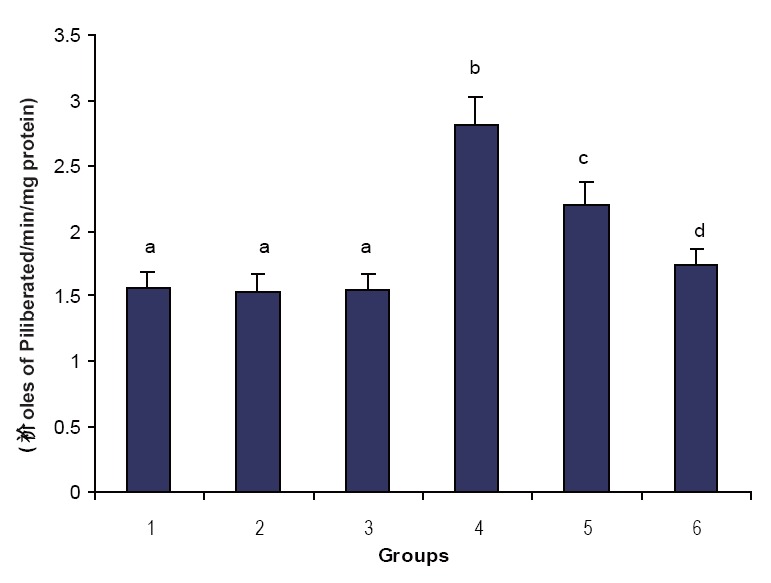
Effect of phytic acid on the activity of calcium dependent adenosine triphosphatase (Mg^2+^ -ATPase) in the heart of normal and isoproterenol (ISO)-induced myocardial infracted rats. Group 1: normal control; Groups 2-3: Normal + phytic acid (25 and 50 mg/kg); Group 4: ISO control (85 mg/kg); Group 5-6: phytic acid (25 and 50 mg/kg) + ISO. Each value is mean ± S.D. for 6 rats in each group. Columns not sharing a common letter (a, b, c and d) differ significantly with each other (*p*<0.05, DMRT).

Figures 1, 2 & 3 illustrate the effect of phytic acid on the activities of Na^+^/K^+^-ATPase, Ca^2+^ and Mg^2+^-ATPases in normal and ISO- induced rats. Na^+^/K^+^-ATPase was decreased significantly and the activities of Ca^2+^ and Mg^2+^-ATPases were increased significantly in the heart of ISO-induced rats when compared to normal control rats. Phytic acid (25 and 50 mg/kg) pretreatment to ISO-induced rats increased significantly the activity of Na^+^K^+^-ATPase and decreased the activities of Ca^2+^ and Mg^2+^-ATPases in the heart when compared to ISO –alone induced rats.

The levels of glycoprotein components (hexose, hexosamine, fucose and sialic acid) in serum of normal and ISO-induced rats is shown is Table 1. Significantly increased levels of glycoprotein components were observed in serum of ISO-induced rats when compared with normal control rats. Pretreatment with phytic acid (25 and 50 mg/kg) decreased significantly the levels of glycoprotein components in serum of ISO-induced rats when compared with ISO-alone induced rats.

**Table 1:** Effect of phytic acid on the levels of glycoprotein components in serum of normal and isoproterenol (ISO)- induced myocardial infacted rats

Groups	Hexose (mg/dL)	Hexosamine (mg/dL)	Fucose (mg/dL)	Sialic acid (mg/dL)

Normal control	115.7 ± 8.20^a^	22.59 ± 1.46^a^	24.53 ± 1.44^a^	30.13 ± 1.77^a^
Normal + phytic acid (25 mg/kg)	114.7 ± 7.67^a^	22.53 ± 1.25^a^	24.19 ± 1.23^a^	29.01 ± 1.65^a^
Normal + phytic acid (50 mg/kg)	113.7 ± 6.53^a^	22.16 ± 1.37^a^	23.75 ± 1.64^a^	29.64 ± 1.61^a^
ISO (85 mg/kg) control	217.4 ± 12.65^b^	34.84 ± 2.13^b^	39.52 ± 1.87^b^	42.61 ± 2.13^b^
Phytic acid (25 mg/kg) + ISO	148.8 ± 10.67^c^	26.75 ± 0.61^c^	30.71 ± 2.18^c^	35.71 ± 1.40^c^
Phytic acid (50 mg/kg) + ISO	133.2 ± 8.64^d^	24.06 ± 0.97^d^	27.91 ± 1.98^d^	31.65 ± 2.34^d^

Each value is mean ± S.D. for 6 rats in each group. Values not sharing a common superscript (a-d) differ significantly with each other (*P*<0.05, DMRT).

Table 2 shows the concentration of glycoprotein components (hexose, hexosamine, fucose and sialic acid) in the heart of normal and ISO-induced rats. The concentration of glycoprotein components were increased significantly in the heart of ISO-induced rats when compared with normal control rats. Phytic acid (25 and 50 mg/kg) pretreatment to ISO-induced rats significantly decreased the concentration of these glycoprotein components in the heart when compared with ISO-alone rats.

**Table 2:** Effect of phytic acid on the levels of glycoprotein components in the heart of normal and isoproterenol (ISO)- induced rats

Groups	Hexose (mg/g defatted tissue)	Hexosamine (mg/g defatted tissue)	Fucose (mg/g defatted tissue)	Sialic acid (mg/g defatted tissue)

Normal control	134.2 ± 6.21^a^	4.26 ± 0.12^a^	23.12 ± 1.21^a^	35.31 ± 2.11^a^
Normal+phytic acid (25mg/kg)	133.2 ± 7.71^a^	4.02 ± 0.21^a^	22.09 ± 0.79^a^	35.12 ± 1.75^a^
Normal +phytic acid (50 mg/kg)	133.1 ± 6.25^a^	4.12 ± 0.32^a^	21.98 ± 1.26^a^	35.01 ± 1.69^a^
ISO (85 mg/kg) control	180.76 ± 11.21^b^	7.12 ± 0.26^b^	31.24 ± 2.01^b^	50.21 ± 3.12^b^
Phytic acid (25 mg/kg)+ISO	153.2 ± 10.34^c^	5.41 ± 0.23^c^	27.19 ± 2.11^c^	41.43 ± 3.12^c^
Phytic acid (50 mg/kg)	142.3 ± 9.98^d^	5.11 ± 0.41^d^	25.02 ± 1.56^d^	37.23 ± 2.37^d^

Each value is mean ± S.D. for 6 rats in each group. Values not sharing a common superscript (a, b, c and d) differ significantly with each other (*p*<0.05, DMRT).

## DISCUSSION

Isoproterenol induced MI serves as a standardized model to study the beneficial effects of many drugs and cardiac function. ISO, in large dose induces morpho- logical and functional alterations in the heart leading to myocardial necrosis (28). It also produces excessive production of free radicals resulting from oxidative metabolism of catecholamines.

Although Table 2 shows the concentration of glycoprotein components (hexose, hexosamine, fucose and sialic acid) in the heart of normal and ISO-induced rata. The concentration of glycoprotein components were increased significantly in the heart of ISO-induced rats when compared with normal control rats. Phytic acid (25 and 50 mg/kg) pretreatment to ISO-induced rats significantly decreased the concentration of these glycoprotein components in the heart when compared with ISO-alone rats.

Cardiotoxicity occurs primarily via adrenoceptor activa- tion (29), there is increasing evidence that it may also occur through oxidative mechanisms (30). Dhalla *et al*. (31) have reported that excess catecholamines affect calcium transport mech- anism primarily via oxidation reactions involving free radical mediated damage and antioxidants may be indi- cated for stress-induced heart disease.

Determination of membrane associated enzyme activities like ATPases indicate the alterations in membrane under pathological conditions. ATPases are intimately associated with the plasma membrane and participates in the energy requiring translocation of sodium, potassium, calcium and magnesium (9). The inhibition of sodium potassium dependent adenosine triphosphatase (Na^+^/K^+^-ATPase) can activate the Na^+^-Ca^2+^ exchange mechanism in the myocardium. This Na^+^-Ca^2+^ exchange mechanism may play a vital role in regulating the cellular calcium levels (10). Calcium dependent adenosine triphosphatase (Ca^2+^-ATPase) is the major active calcium transport protein responsible for the maintenance of normal intracellular calcium levels in a variety of cell types.

In this study, we observed a decrease in the activity of Na^+^/K^+^-ATPase and increase in the activities of Ca^2+^ and magnesium dependent adenosine triphosphatase (Mg^2+^-ATPase) in ISO-induced rats. Since Na^+^/K^+^-ATPase is a ‘SH’ group containing enzyme and is lipid dependent, the inactivation of Na^+^/K^+^-ATPase could be due to enhanced lipid peroxidation by free radicals on ISO-induction (32). Enhanced Ca^2+^-ATPase observed in ISO-induced rats is due to adenylate cyclase activation by ISO. Calcium overload in the myocardial cells during ischemia activate the Ca^2+^ dependent ATPase of the membrane depleting high energy phosphate stores thereby indirectly inhibiting the Na^+^ and K^+^ transport as well as the activity of Na^+^/K^+^-ATPase (32). Pretreatment with phytic acid increased the activity of Na^+^/K^+^-ATPase and decreased the activities of Ca^2+^ and Mg^2+^-ATPases in ISO-induced rats. This could be due to the ability of phytic acid to protect the ‘SH’ groups from the oxidative damage through the inhibition of peroxidation of membrane lipids. This effect might be due to the membrane stabilizing action of phytic acid.

The functions of glycoproteins in stabilizing the tissue may be involved in maintaining the structural stability of collagen fibrils. Glycoproteins are important components of intracellular matrix, cell membrane and membranes of the subcellular organelles (33). In this study, we have observed a significant increase in the levels of hexose, hexosamine, fucose and sialic acid in serum and the heart of ISO-induced rats. Lindberg *et al.* (34) have reported that the levels of serum and the heart glycoproteins were increased significantly in CVD. Mathew *et al*. (35) also reported similar changes in serum and the heart glycoproteins in ISO-induced myocardial infarcted rats.

The elevation in the levels of serum glycoprotein components might be due to secretion from cell membrane glycoconjugates into the circulation (36). The observed increase in the levels of glycoprotein in ISO-induced rats may also be due to increased deposition of macromolecular components, which is a physiological adjustment to the pathological process. Judd and Wexler (37) suggested that glycoproteins are involved in the myocardial necrosis and repair. phytic acid pretreatment decreased the levels of glycoproteins in serum and the heart in ISO-induced MI in rats. This could be due to membrane stabilizing and antioxidant property of phytic acid, which ultimately helps to maintain the levels of glycoprotein components in ISO-induced rats.

## CONCLUSION

Our study shows that phytic acid exhibits cardioprotec- tive effect in ISO-induced MI rats by maintaining the membrane bound enzymes and glycoproteins levels to near normal. The cardioprotective effect of phytic acid could be due to the membrane stabilizing and antioxidant properties of phytic acid.
